# Unusual symptomatic inclusion cysts in a newborn: a case report

**DOI:** 10.1186/1752-1947-8-314

**Published:** 2014-09-21

**Authors:** Roberta Marini, Nicolae Chipaila, Annalisa Monaco, Domenico Vitolo, Gian Luca Sfasciotti

**Affiliations:** 1 Department of Oral and Maxillo-Facial Sciences, “Sapienza” University of Rome, Via Caserta, 6 - 00161 Rome, Italy; 2 Department of Life, Health and Environmental Sciences, University of L’Aquila, L’Aquila, Italy; 3 Department of Pathology, “Sapienza” University of Rome, Rome, Italy

**Keywords:** Dental lamina cyst, Epstein’s pearls, Mucosal cyst, Newborn inclusion cyst

## Abstract

**Introduction:**

Dental lamina cysts are cysts that occur as white or pink small nodules, often multiple, approximately 1 to 3mm in diameter. They are typically located on the midpalatine raphe and less frequently on the maxillary and mandibular alveolar mucosa; in the latter case these can be appear to be neonatal teeth.

On microscopic examination, these lesions show a stratified squamous epithelium (two to three cell layers); it is possible to find protein, keratin and/or exfoliated epithelial cells in the lumen of the lesions.

Neonatal cysts usually show no particular symptoms. They are associated with an excellent prognosis because they regress spontaneously within a few weeks and are not associated to any complications. However, if pain, bleeding or other symptoms occur, a surgical excision is required.

**Case presentation:**

In this paper, we present an anomalous case of symptomatic dental lamina cyst which affected a 60-day-old male Caucasian newborn. The surgical treatment was elective in this case and 6-month follow-ups were mandatory.

**Conclusions:**

We can underline the successful predictability of the surgical approach; however, we consider that the treatment choice should take place in the light of medical history and clinical considerations, and always be evaluated on a case-by-case basis. Further studies and reviews in this field should be performed in order to suggest guidelines for clinicians, although these cases are rare.

## Introduction

Many features of a baby’s mouth are unique and peculiar to this development period, such as gingival cysts, inclusion cysts and natal teeth [1-4].

In 1967, according to histogenesis and location in the oral cavity, Fromm [5] classified oral cysts as Epstein’s pearls, Bohn’s nodules and dental lamina cysts. This classification was based on their location and histology: all neonatal cysts are keratin-filled nodules but Epstein’s pearls are predominantly located along the midpalatine raphe and probably derive from residual epithelial cells arising from embryonic palatine processes. Bohn’s nodules derive from palatal salivary glands’ structures and their most common site is the palate. Dental lamina cysts occur as white or pink small nodules, often multiple, approximately 1 to 3mm in diameter. Dental lamina cysts are typically located on the midpalatine raphe and less frequently on the maxillary and mandibular alveolar mucosa; in the latter case these can appear to be neonatal teeth. It is probable that these lesions originate from remnants of the dental lamina.

This case report describes the unusual clinical presentation and management of multiple oral cysts in a newborn baby.

## Case presentation

**Figure 1:**
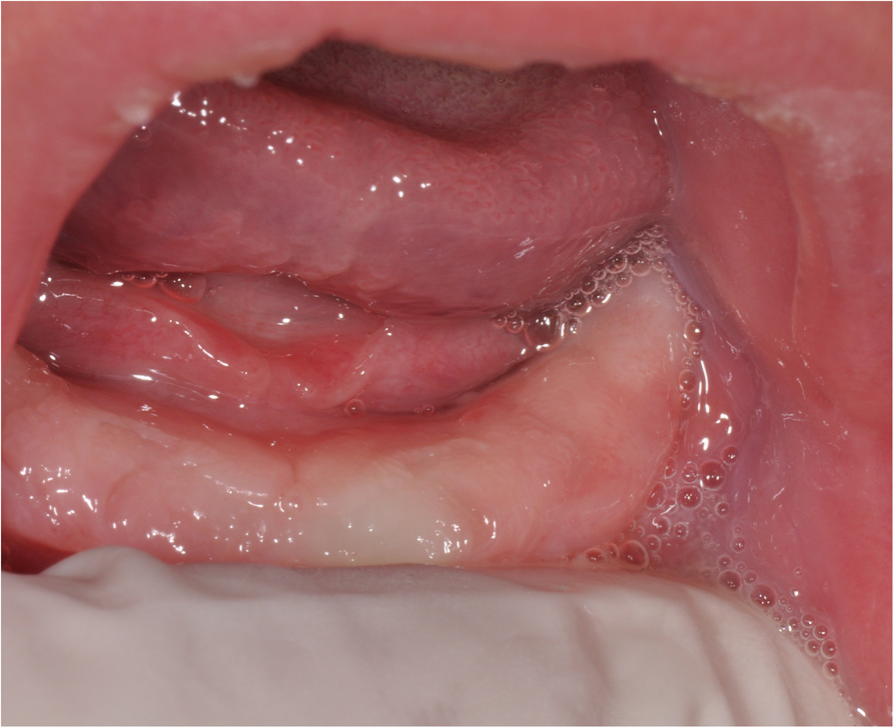
Intraoral examination of mandible.

A Caucasian 60-day-old male newborn baby came to our observation. His parents complained that he had bleeding gums in his lower and upper jaws, pain in sucking and consequent difficulties in feeding. He was full-term born and his weight at birth was 2900g. His past medical history was not significant.No noticeable findings were recorded during an extraoral examination. The intraoral examination revealed multiple whitish small masses localized on the alveolar ridge of his maxilla and mandible. His mandibular alveolar ridge showed small nodular lesions in the region of the right deciduous canine and left deciduous canine and first molar (Figure 1). The size of these masses varied from 3 to 4mm in diameter.

A surgical removal of the lesions was planned in order to solve feeding problems and bleeding.

**Figure 2:**
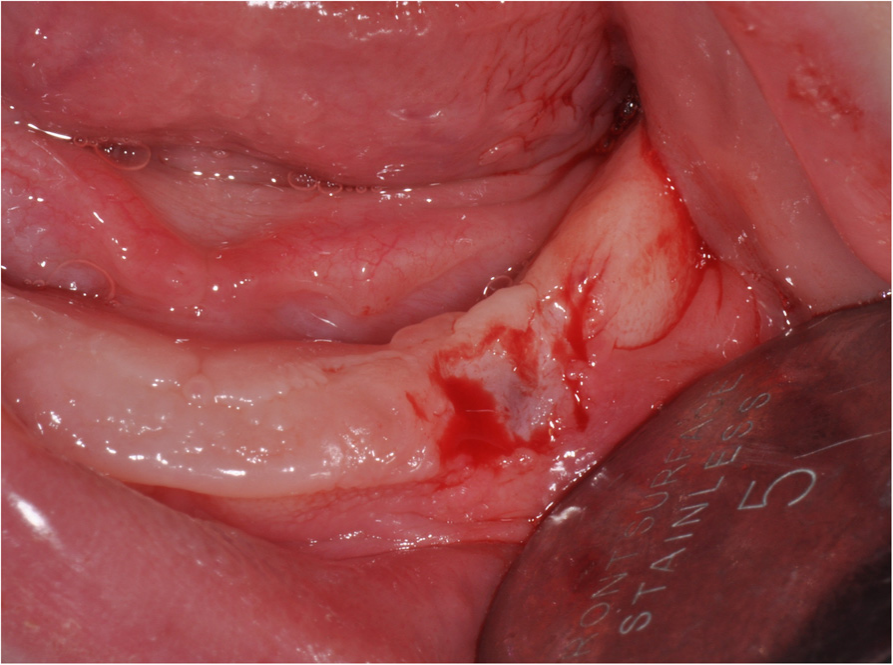
Excision of lesions on the left mandibular gum.

**Figure 3:**
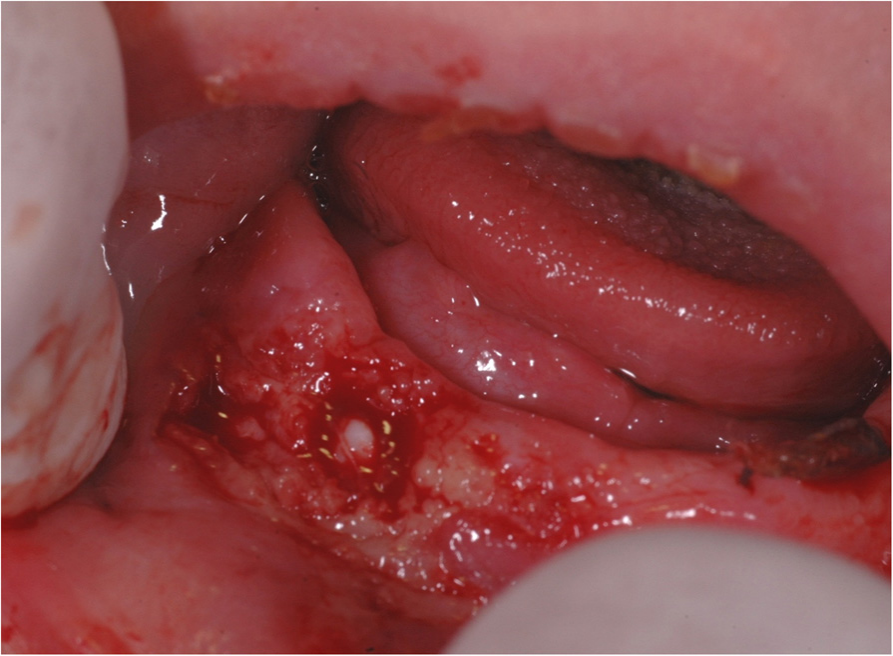
Excision of lesions on the right mandibular gum.

**Figure 4:**
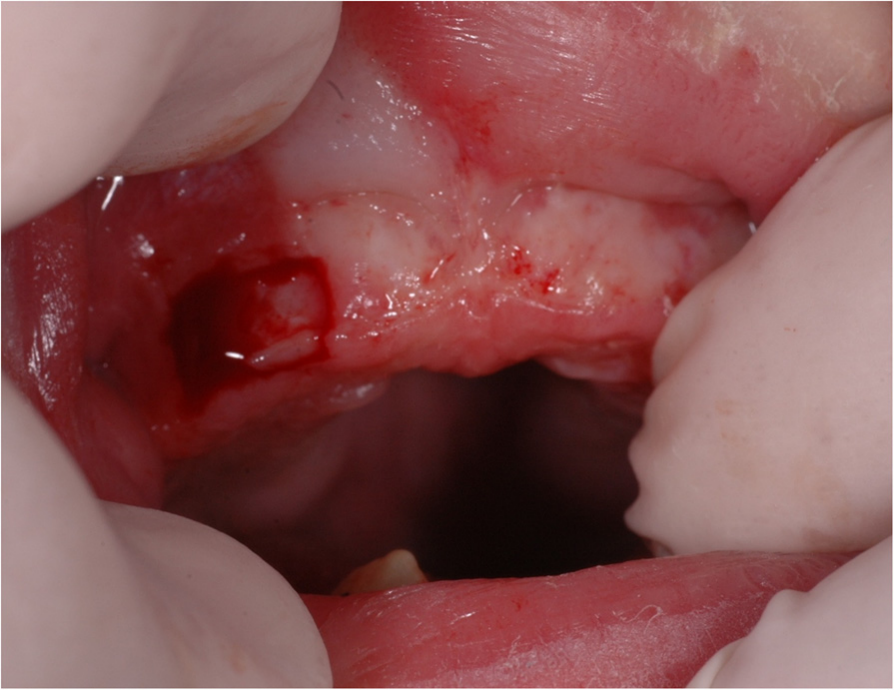
Excision of lesions on the maxillary gum.

The excisions were performed in different sessions in order to limit the postoperative discomfort to the baby. All oral surgeries were performed under local anesthesia (mepivacaine 3% without adrenalin) with a 15C scalpel blade. After excision of the cysts a sponge of fibrin was used and the wounds were sutured with resorbable polyglactin suture (Vicryl 4-0; Ethicon, Johnson & Johnson, New Brunswick, New Jersey, USA) that was removed after 10 days.The first surgery involved the excision of two lesions on his left mandibular gum (Figure 2).The second surgical session was made after 15 days. The excision of his right mandibular lesions revealed a neonatal tooth. In this step the surgeon decided not to remove it because the tooth incisal margin was below the gingival level and it would not create feeding problems (Figure 3). A follow-up visit 30 days after the surgery showed the neonatal tooth eruption and a new maxillary lesion in the deciduous first molar region. A new surgical cyst excision was necessary and, in the same session, the neonatal tooth extraction was performed in order to allow the baby breastfeeding without hurting the mother (Figure 4).

**Figure 5:**
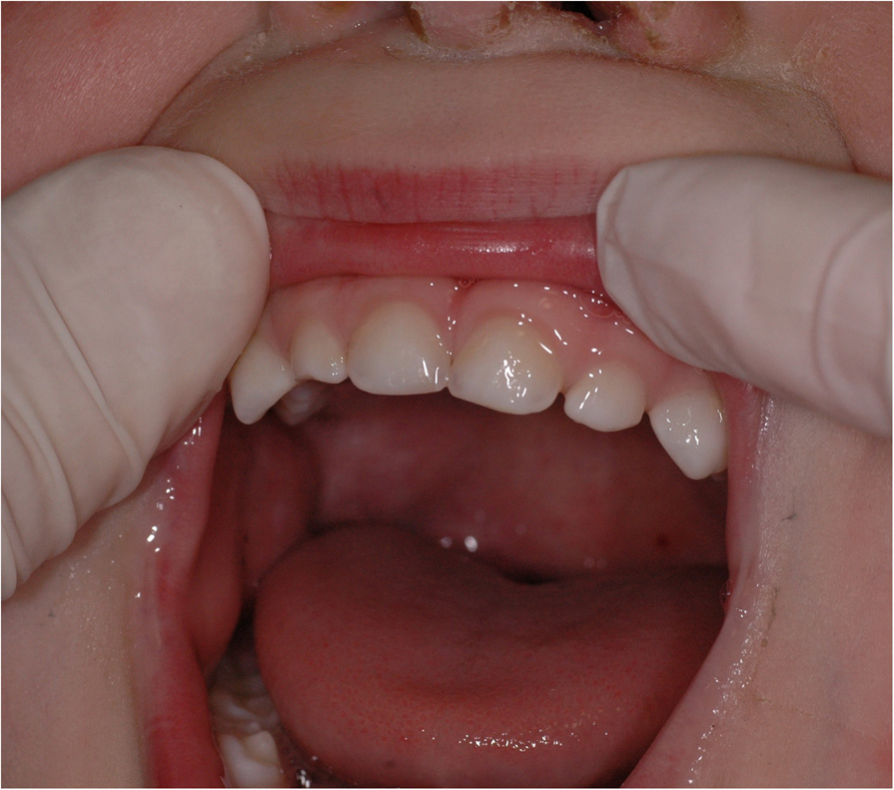
A clinical examination of the patient at the age of 2 years showing normal dentition.

**Figure 6:**
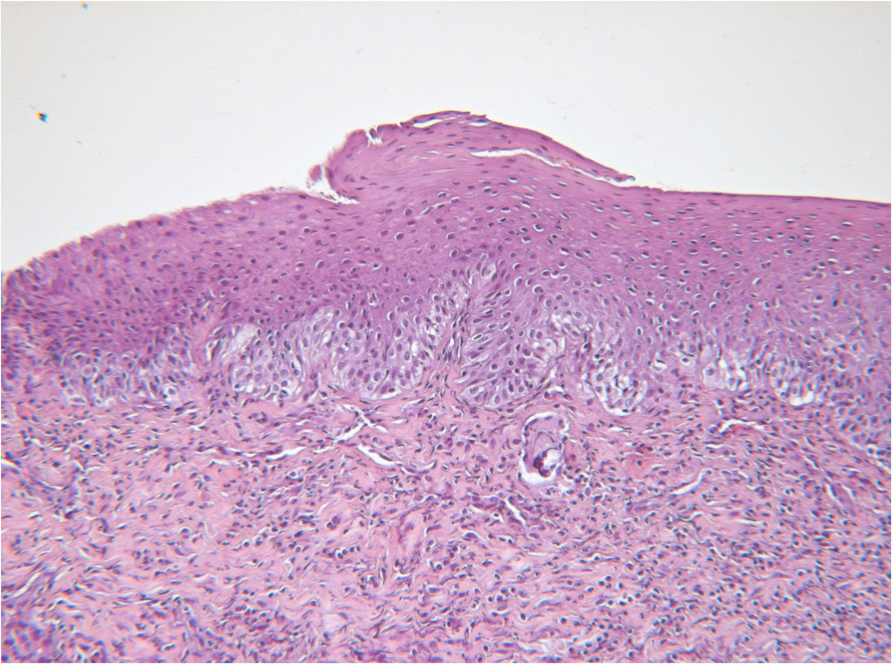
Biopsy specimen of first removed cysts: histological examination showed small islands of odontogenic epithelium (remnants of dental lamina). Hematoxylin and eosin, ×200.

The obtained specimens were fixed in 10% formalin solution and submitted for histological examination.Periodic recall visits were advised to verify the absence of new lesions and to monitor the developing dentition (Figure 5).All three histological examinations revealed that the characteristics of the lesions were compatible with the diagnosis of dental lamina cysts: they were lined with keratinizing, stratified squamous epithelium, developing from islands of basophilic epithelium, which were interpreted as remnants of the dental lamina (Figure 6).

## Discussion

The dental lamina cyst, also known as gingival cyst of newborn, is considered a true cyst because it is lined by thin epithelium with a lumen usually filled with desquamated keratin and occasionally inflammatory cells [6,7]. These cysts can easily be wrongly diagnosed as natal teeth, especially if they are located in the newborn’s mandibular anterior ridge. They typically appear as multiple nodules along the alveolar ridge in babies. It is believed that fragments of dental lamina remain within the alveolar ridge mucosa after tooth formation and then proliferate to develop these small, keratinized cysts. However, many of them degenerate, reduce in size or are broken spontaneously in the oral cavity within 2 weeks to 5 months of postnatal life [8-11].

It is more difficult to elucidate the method of their disappearance. In a study of 32 human fetal heads from 8 to 22 weeks of fetal age, not more than 20 midpalatal raphe cysts were found in any fetus by week 14, and they did not increase in frequency with time [12]. The authors suggested that as the cysts developed, the epithelium differentiated, fused with the oral epithelium and the contents discharged. Some cysts may produce keratin, enlarge, extend to the surface and rupture during the first few months after birth [12-14].

As regards the prevalence of oral cysts in newborns, George *et al*. [6] in a study of 1038 newborn Indian babies, found that gingival cysts were present in 143 neonates (13.8%): 112 (10.8%) on the alveolar region, 19 (1.8%) palatally and 12 (1.2%) on both regions; moreover, in 5 cases, gingival cysts were present on the mandibular ridge. Epstein’s pearls were present in 365 newborn (35.2%), and were located in the midline within the median raphae of the hard palate in the maxilla. Bohn’s nodules were present in 492 newborn (47.4%), which were located at the junction of the hard and soft palate, adjacent to the midpalatal raphae. Fromm [5] reported cysts in 75.9% of 1367 neonates less than 48-hours old and Cataldo and Berkman [1], in a study of 209 neonates between 1 and 5 days of age, reported that the most common location for the cysts was along the median palatal raphe (65.1%) and the least common site was the mandibular alveolar mucosa (9.9%). Jorgensen *et al*. [7] found palatal cysts in 73% of 596 White children in the USA and alveolar cysts in 53.5%. Cetinkaya *et al*. [2], through an oral examination of 2021 newborn Turkish children, registered that palatally located oral mucosal cysts were the most common findings (29%).

Whereas some authors reported a female predilection [10,15], others found no gender differences [7]. Moreover, in some surveys [7,11,15] an increased incidence has been noticed in Caucasian as opposed to Negro children; our patient was a Caucasian newborn.

Donley and Nelson [3] demonstrated that oral cysts are correlated with gestational age and weight at birth. They are less frequent in preterm newborns or those with weight at birth of less than 2500g. A greater frequency of submucous cysts with less clinical prevalence or greater difficulty in clinical exploration of preterm infants could explain these differences. Our patient was a full-term baby with weight at birth of 2900g.

The particularity of this case is due to two unusual features that occur in this patient: the presence of symptoms and the different onset time of the lesions. In fact, all the cysts showed spontaneous bleeding and tenderness to palpation. In addition, during the first observation only the mandibular cysts were present; after 30 days the maxillary lesion also appeared.

The treatment options have been: no treatment and follow up, marsupialization or surgical extraction. In this case the surgical treatment was mandatory because of the inability of the baby to feed properly.

The role of histopathology in establishing the final diagnosis is not essential because the treatment was required by the symptomatology of the newborn; however, it is important to prevent any misdiagnosis, such as hemangioma, melanoma, unicystic ameloblastoma, keratinizing cystic odontogenic tumor and mucocele [16-18].

Inclusion cysts may be associated to natal or neonatal teeth like in this case. When natal or neonatal teeth interfere with feeding, have high mobility, and/or are poorly developed, there is the indication to remove them [15].

## Conclusions

The present case of inclusion cysts was successfully managed by the surgical excision of the lesions and the extraction of the neonatal tooth, in order to promote the baby’s natural breastfeeding, without hurting his mother. The parents were well informed about the pathology and its implications. After surgery, both the child and his mother had no complications during feeding; normal eruption of primary teeth occurred by the 6th month. Although for this patient surgical excision of the lesions was the ideal approach, in our opinion the treatment choice must be carefully evaluated on a case-by-case basis after an adequate analysis of clinical findings. Moreover, further studies (mainly randomized controlled trials) on this topic should be performed in order to draw up guidelines for clinicians and oral surgeons, although these cases are quite rare.

## Consent

Written informed consent was obtained from the patient’s parents for publication of this case report and any accompanying images. A copy of the written consent is available for review by the Editor-in-Chief of this journal.

## Competing interests

The authors declare that they have no competing interests.

## Authors’ contributions

RM conceived the study and drafted the manuscript, NC and AM participated in its design and coordination, DV performed the histopathological analysis and GLS performed the surgery. All authors read and approved the final manuscript.

## References

[B1] Cataldo E Berkman MD Cyst of the oral mucosa in newborns Amer J Dis Child 1968 116 44 48 5657354 10.1001/archpedi.1968.02100020046006

[B2] Cetinkaya M Oz FT Orhan AI Orhan K Karabulut B Karabulut DCC Ilk O Prevalence of oral abnormalities in a Turkish newborn population Int Dent Jour 2011 61 90 100 10.1111/j.1875-595X.2011.00020.x PMC9374825 21554278

[B3] Donley CL Nelson LP Comparison of palatal and alveolar cysts of the newborn in premature and full term infants Pediatr Dent 2000 22 321 324 10969441

[B4] Freudenberger S Santos Diaz MA Bravo JM Sedano HO Intraoral findings and other developmental conditions in Mexican neonates J Dent Child 2008 75 3 280 286 19040815

[B5] Fromm A Epstein pearls, Bohn’s nodules and inclusion cyst of the oral cavity J Dent Child 1967 34 275 287 5342399

[B6] George D Bhat SS Hedge SK Oral findings in newborn children in and around Mangalore, Karnataka State, India Med Princ Pract 2008 17 385 389 10.1159/000141502 18685278

[B7] Jorgensen RJ Shapiro SD Salinas CF Levin LS Intraoral findings and anomalies in neonates Pediatrics 1982 69 5 577 582 7079011

[B8] Kumar A Grewal H Verma M Dental lamina cyst of newborn: a case report J Indian Soc Pedod Prev Dent 2008 26 4 175 176 10.4103/0970-4388.44039 19008628

[B9] Mohta A Sharma M Congenital oral cysts in neonates: report of two cases Oral Surg Oral Med Oral Radiol Endod 2006 102 5 e36 e38 10.1016/j.tripleo.2006.03.024 17052622

[B10] Monteagudo B Labandiera J Cabanillas M Acevedo A Leon-Muinos E Toribio J Prevalence of milia and palatal and gingival cysts in Spanish newborns Pediatr Dermatol 2012 29 3 301 305 10.1111/j.1525-1470.2011.01433.x 21995277

[B11] Monteleone L McLellan MS Epstein’s pearls (Bohn’s nodules) of palate J Oral Surg 1964 22 301 304 14155470

[B12] Moreillon MC Schroeder HE Numerical frequency of epithelial abnormalities, particularly microkeratocysts, in the developing human oral mucosa Oral Surg Oral Med Oral Pathol 1982 53 44 55 10.1016/0030-4220(82)90485-6 6172766

[B13] Navas RMA Mendoza MGM Leonardo MR Silva RAB Herrera HW Herrera HP Congenital eruption cyst: a case report Braz Dent J 2010 21 3 259 262 10.1590/S0103-64402010000300015 21203711

[B14] Paula JD Dezan CC Frossard WT Walter LR Pinto LM Oral and facial inclusion cysts in newborns J Clin Pediatr Dent 2006 31 127 129 17315809 10.17796/jcpd.31.2.rw3h853m3rk242q0

[B15] Rao RS Mathad SV Natal teeth: Case report and review of literature J Oral Maxillofac Pathol 2009 13 41 46 10.4103/0973-029X.44574 21886998 PMC3162856

[B16] Richard BM Qiu CX Ferguson MWJ Neonatal palatal cysts and their morphology in cleft lip and palate Brit Journ Plast Surg 2000 53 555 558 10.1054/bjps.2000.3410 11000069

[B17] Woldenberg Y Goldstein J Bodner L Eruption cyst in the adult – a case report Int J Oral Maxillofac Surg 2004 33 804 805 10.1016/j.ijom.2003.10.018 15556332

[B18] Sridevi K Nandan SR Ratnakar P Srikrishna K Pavani Vamsi B Residual cyst associated with calcifications in an elderly patient J Clin Diagn Res 2014 8 2 246 249 10.7860/JCDR/2014/7593.4072 PMC3972577 24701547

